# Text-Based Illness Monitoring for Detection of Novel Influenza A Virus Infections During an Influenza A (H3N2)v Virus Outbreak in Michigan, 2016: Surveillance and Survey

**DOI:** 10.2196/10842

**Published:** 2019-04-26

**Authors:** Rebekah J Stewart, John Rossow, Seth Eckel, Sally Bidol, Grant Ballew, Kimberly Signs, Julie Thelen Conover, Erin Burns, Joseph S Bresee, Alicia M Fry, Sonja J Olsen, Matthew Biggerstaff

**Affiliations:** 1 Influenza Division National Center for Immunization and Respiratory Diseases Centers for Disease Control and Prevention Atlanta, GA United States; 2 Epidemic Intelligence Service Centers for Disease Control and Prevention Atlanta, GA United States; 3 Epidemiology Elective Program, Division of Scientific Education and Professional Development Center for Surveillance, Epidemiology, and Laboratory Services Centers for Disease Control and Prevention Atlanta, GA United States; 4 College of Veterinary Medicine University of Georgia Athens, GA United States; 5 Michigan Department of Health and Human Services Lansing, MI United States; 6 Compliant Campaign Scottsdale, AZ United States; 7 Michigan State University Extension East Lansing, MI United States

**Keywords:** influenza, surveillance, novel, agricultural, fairs, texting

## Abstract

**Background:**

Rapid reporting of human infections with novel influenza A viruses accelerates detection of viruses with pandemic potential and implementation of an effective public health response. After detection of human infections with influenza A (H3N2) variant (H3N2v) viruses associated with agricultural fairs during August 2016, the Michigan Department of Health and Human Services worked with the US Centers for Disease Control and Prevention (CDC) to identify infections with variant influenza viruses using a text-based illness monitoring system.

**Objective:**

To enhance detection of influenza infections using text-based monitoring and evaluate the feasibility and acceptability of the system for use in future outbreaks of novel influenza viruses.

**Methods:**

During an outbreak of H3N2v virus infections among agricultural fair attendees, we deployed a text-illness monitoring (TIM) system to conduct active illness surveillance among households of youth who exhibited swine at fairs. We selected all fairs with suspected H3N2v virus infections. For fairs without suspected infections, we selected only those fairs that met predefined criteria. Eligible respondents were identified and recruited through email outreach and/or on-site meetings at fairs. During the fairs and for 10 days after selected fairs, enrolled households received daily, automated text-messages inquiring about illness; reports of illness were investigated by local health departments. To understand the feasibility and acceptability of the system, we monitored enrollment and trends in participation and distributed a Web-based survey to households of exhibitors from five fairs.

**Results:**

Among an estimated 500 households with a member who exhibited swine at one of nine selected fairs, representatives of 87 (17.4%) households were enrolled, representing 392 household members. Among fairs that were ongoing when the TIM system was deployed, the number of respondents peaked at 54 on the third day of the fair and then steadily declined throughout the rest of the monitoring period; 19 out of 87 household representatives (22%) responded through the end of the 10-day monitoring period. We detected 2 H3N2v virus infections using the TIM system, which represents 17% (2/12) of all H3N2v virus infections detected during this outbreak in Michigan. Of the 70 survey respondents, 16 (23%) had participated in the TIM system. A total of 73% (11/15) participated because it was recommended by fair coordinators and 80% (12/15) said they would participate again.

**Conclusions:**

Using a text-message system, we monitored for illness among a large number of individuals and households and detected H3N2v virus infections through active surveillance. Text-based illness monitoring systems are useful for detecting novel influenza virus infections when active monitoring is necessary. Participant retention and testing of persons reporting illness are critical elements for system improvement.

## Introduction

Novel influenza A viruses are different from currently circulating human influenza A (H1 and H3) viruses and have the potential to cause a pandemic if viruses gain the capacity to infect and transmit efficiently from person to person and cause clinical illness in humans [1]. In the United States, human infection with a novel influenza A virus is nationally notifiable [1]; swine are the primary source of reported novel influenza A virus infections in humans, and the vast majority of persons are infected after swine exposure at an agricultural fair [2-9]. Some influenza A viruses are endemic pathogens in swine populations [10-13] and swine can be infected without displaying clinical signs of illness [14]. Monitoring for novel influenza virus infections in humans is important to quickly identify viruses with pandemic potential and to speed implementation of an effective public health response. However, traditional forms of active monitoring for illness (eg, through daily phone calls to persons with possible exposure) can be very labor intensive for health department staff; during an outbreak, the need for monitoring may overwhelm a health department’s ability to respond in a timely manner. The US Centers for Disease Control and Prevention (CDC) recommends that people participating in avian influenza outbreak response efforts be monitored during the response and for 10 days after their last possible exposure [15], which can result in a substantial number of responders under monitoring per state at any given time. For example, during an avian influenza outbreak in 2014-2015 in Minnesota, USA, public health officials conducted active postexposure symptom monitoring for 459 responders by making daily phone calls for 10 days (Karen Martin, epidemiologist in Minnesota, personal communication). Short message service (SMS) text messaging has been used previously in research [16] and outbreak settings [17] to facilitate the monitoring of multiple individuals for respiratory illness.

To assist US states with monitoring individuals at potential risk of a novel influenza virus infection, CDC, in coordination with the National Association of County and City Health Officials (NACCHO) and Compliant Campaign, developed a text-message illness monitoring (TIM) platform. Although several state health departments had pilot-tested the TIM system, none had previously used it among members of the public during an outbreak. On August 4, 2016, public health officials in Michigan, USA, notified CDC of a laboratory-confirmed influenza A (H3N2) variant (H3N2v) virus infection in a 9-year-old child who had been exhibiting swine at an agricultural fair in Michigan [18,19]. Public health officials in Michigan reported a second infection the next day along with reports of ill swine at a second agricultural fair.

On August 9, 2016, the Michigan Department of Health and Human Services (MDHHS) asked CDC to pilot-test the TIM system to monitor families of individuals exhibiting swine at county fairs and evaluate the functionality during an outbreak. The objectives of the pilot were to determine the ability of the TIM system to enhance detections of H3N2v virus infections and to evaluate the feasibility and acceptability of the TIM system for use in future outbreak investigations of novel influenza viruses.

## Methods

### Enrollment, Retention, and Illness Detection

The details of the H3N2v outbreak have been described previously (see Multimedia Appendix 1) [18]. For the TIM system pilot, we targeted the following:

1. County agricultural fairs that had reported suspected H3N2v infections in the last 10 days during the 2016 H3N2v outbreak.
2. County fairs, to ensure illness follow-up and testing could be coordinated by a single county health department, that started during our enrollment period—August 15 to September 9, 2016—and had large (>50) numbers of swine in exhibit. Large numbers of swine increase the likelihood of swine influenza outbreaks; we chose a number of more than 50 to reach the upper 50th percentile of swine per fair as determined by Bowman and colleagues [20].

**Figure 1:**
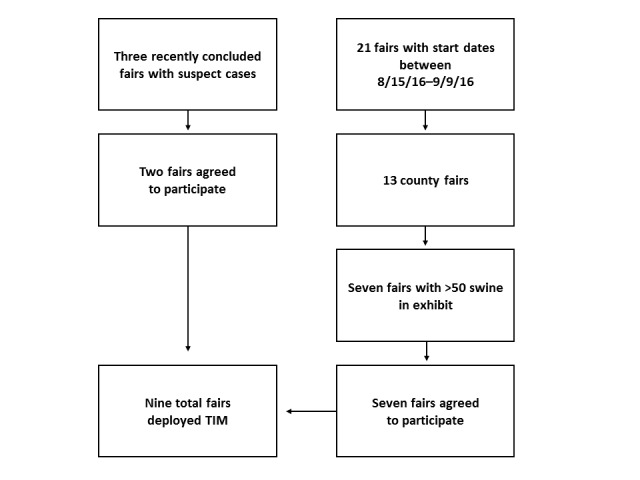
Fair selection. TIM: text-message illness monitoring.

We identified three fairs with suspected H3N2v virus infections and approached fair organizers about participating in the TIM pilot. Of those, organizers of two fairs agreed to participate (see Figure 1). We identified 21 fairs occurring between August 15 and September 9, 2016, from which no human H3N2v infections were reported. Of those, 13 were county fairs and seven of the county fairs had more than 50 exhibition swine. Organizers of these seven fairs agreed to participate in the pilot, bringing the total number of fairs where the TIM system was deployed to nine fairs (see Figure 1).

As studies have shown that young age and direct contact with swine are risk factors for variant influenza virus infection [4,6,9], we contacted coordinators of youth agricultural clubs to invite households of swine exhibitors to participate. For fairs with reported infections, club coordinators sent recruitment information solely through email. For the fairs without reported infections, we attended exhibitor meetings or other fair events to describe variant influenza viruses and the TIM system. We also distributed informational flyers about the TIM system and sent recruitment information by email.

**Figure 2:**
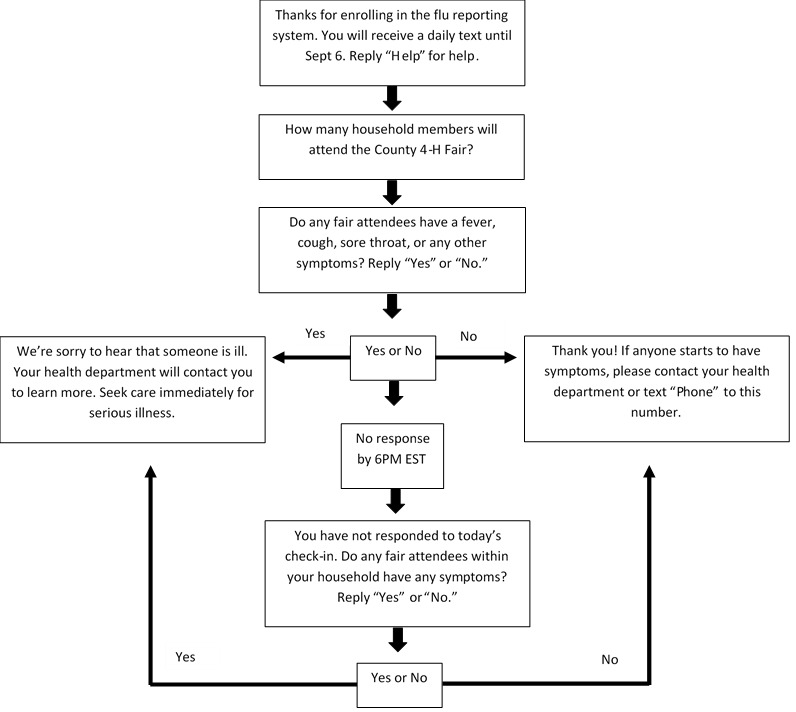
Texting language flow (formal).

**Figure 3:**
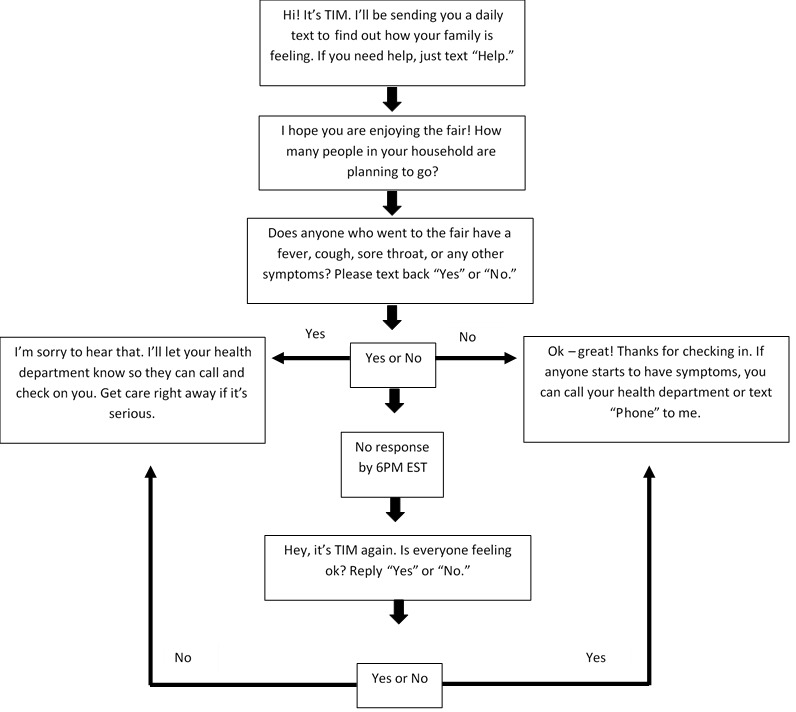
Texting language flow (informal). TIM: text-message illness monitoring.

We assigned each fair a unique texting code word. Parents of swine exhibitors were asked to text in the unique code word using their mobile phone, which would automatically enroll them in the system (see Figures 2 and 3). After texting the code word, enrollees were asked how many household members were attending or planning to attend the county fair. Next, they were asked if any fair attendees had a fever, cough, sore throat, or other symptoms. Respondents who replied “No” were advised to contact the health department if anyone started to have symptoms. Respondents who replied “Yes” were advised that a representative from the health department would contact them to learn more about the illness. If a response was not received by 6 PM, a reminder text was automatically sent. Texts asking about illness were sent daily at noon during the fair and for 10 days following the fair. Because CDC does not recommend a specific monitoring period for persons exposed to variant influenza viruses, we used the monitoring period recommended by CDC for avian influenza responders [21].

“Yes” responses and any response other than “No” (eg, “OK”) generated an immediate email alert to designated local health department staff to contact the respondent and determine if additional follow-up was necessary. Health department staff were asked to refer anyone for respiratory testing who reported any symptoms associated with variant influenza virus infection, including mild symptoms such as runny nose and less common symptoms such as vomiting, and who had swine exposure or contact with a person with a confirmed H3N2v virus infection during the week prior to illness onset [6]. Collected specimens underwent real-time reverse-transcription polymerase chain reaction (rRT-PCR) or genetic sequencing at CDC or the state public health laboratory to confirm H3N2v virus isolation.

We monitored retention of respondents throughout the monitoring period and assessed the impact of reminder texts on daily participation. Respondents could opt out at any time by texting “STOP.” Estimates of user retention were based on formal withdrawals and respondents who ceased responding. To understand the impact of the type of texting language on respondent retention, we randomly assigned fairs to one of two groups where attendees would receive texts with either more formal or less formal language (see Figures 2 and 3). We applied a Poisson regression using generalized estimating equations to examine statistically significant differences in daily respondent counts between the two types of texting language. The threshold for statistical significance was set at *P*<.05.

### Web-Based Survey

We developed a Web survey to evaluate the use and acceptability of the TIM system using Epi Info software, version 7.2.0.1 (Centers for Disease Control and Prevention). The youth program club coordinators distributed a link to the survey by email to all swine exhibitor families after the conclusion of the fair season. Questions included reasons for participating or not participating in the TIM system and likelihood of participating again if given the opportunity.

### Health Department Interviews

We conducted five unstructured interviews with health officials at participating health departments. These were conducted to determine how much time was spent following up on alerts generated by the system and to determine how many respondents with reports of illness sought care and were tested for influenza.

### Human Subjects Determination

This investigation was determined to be part of a public health response; in accordance with federal human subjects’ protection regulations, it was not considered human subjects research. Participation in the survey was voluntary and anonymous.

## Results

### Enrollment, Retention, and Illness Detection

Out of an estimated 500 households contacted at the nine selected fairs, 87 (17.4%) households enrolled, reporting for 392 household members (see Table 1). Household participation rates varied by fair and by enrollment method (range 3%-86%, median 13%).

**Table 1:** Recruitment methods and enrollment by fair.

Fair	Primary recruitment method	Recruitment occurred after fair conclusion	H3N2v^a^ infections associated with fair	Swine exhibitors (N=1052), n (%)	Estimated households^b^ (N=528), n (%)	Peak household enrollment, n/N (%)	Total under TIM^c^ surveillance (N=392), n (%)
Fair A	Meeting	No	No	230 (21.9)	115 (21.8)	21/115 (18.3)	92 (23.5)
Fair B	Email	Yes	Yes	200 (19.0)	100 (18.9)	11/100 (11.0)	54 (13.8)
Fair C	Local health department visit	No	No	125 (11.9)	63 (11.9)	3/63 (5.0)	13 (3.3)
Fair D	Meeting	No	No	122 (11.6)	61 (11.6)	7/61 (11.4)	31 (7.9)
Fair E	Email	Yes	No	120 (11.4)	60 (11.4)	8/60 (13.3)	40 (1.0)
Fair F	Local health department visit	No	No	75 (7.1)	38 (7.2)	1/38 (2.6)	4 (1.0)
Fair G	Meeting	No	No	70 (6.7)	35 (6.6)	30/35 (85.7)	132 (33.7)
Fair H	Flyers	No	No	55 (5.2)	28 (5.3)	1/28 (3.6)	4 (1.0)
Fair I	Flyers	No	No	55 (5.2)	28 (5.3)	5/28 (17.9)	22 (5.6)
Total	N/A^d^	N/A	N/A	1052 (100)	528 (100)	87/528 (16.5)	392 (100)

^a^Influenza A (H3N2) variant.

^b^Derived by dividing the number of reported swine exhibitors by 2, based on experts’ estimates of the maximum likely number of swine exhibitors per household.

^c^TIM: text-message illness monitoring.

^d^N/A: not applicable.

**Figure 4:**
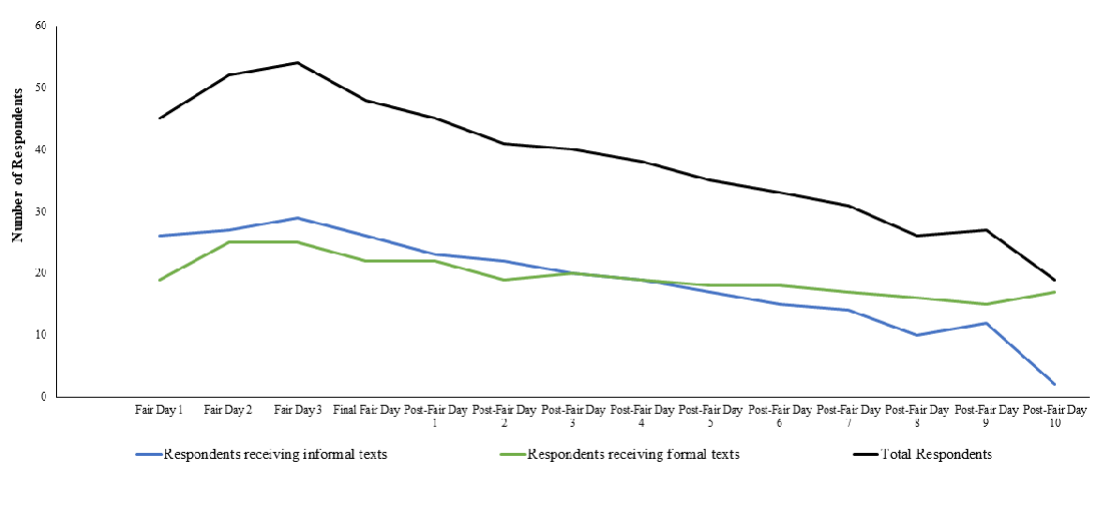
Respondent retention throughout monitoring period: total and by texting language type.

The number of household respondents for ongoing fairs peaked at 54 respondents on the third day of the fair; after that, the number steadily declined throughout the rest of the monitoring period (see Figure 4). Approximately 22% (19/87) of households remained enrolled and responded at the end of the 10-day monitoring period; 54% (47/87) made a report every day they were enrolled. Retention of respondents receiving informal texts declined faster than those receiving the formal texting language (*P*<.01); 7% (2/29) of respondents receiving the informal texts remained enrolled through the end of the monitoring period (see Figure 4). The initial daily text generated 83% of all responses, while 17% of responses followed the second (reminder) text.

Of the 392 persons who were actively monitored, illness was reported for 22 (5.6%) through the TIM system. Of those 22, 9 (41%) sought care; 5 out of the 9 that sought care (56%) had a specimen tested and 2 out of those 5 (40%) tested positive for H3N2v virus, representing 17% (2/12) of all H3N2v virus infections detected in Michigan during this outbreak [18]. Of the 13 symptomatic persons who did not seek care, 2 (15%) could not be reached by the health department, 1 (8%) was instructed not to seek care by the county medical director, 1 (8%) declined seeking care due to the cost, and 1 (8%) declined because another diagnosis was believed to be more likely; the other 8 (62%) did not provide a reason.

### Web-Based Survey

Among five fairs that distributed the survey to swine exhibitor families through an estimated 500 email addresses, 70 households (14.0%) who exhibited swine responded. Respondents from 16 out of 70 (23%) households reported participating in TIM. Among the 15 respondents who answered the question regarding motivation for participation, 11 (73%) participated because it was recommended by the fair and 12 (80%) indicated that they would participate again. Among 50 respondents who reported not participating in TIM and responded to the question, 34 (68%) said it was because they had not heard about it, 7 (14%) said they did not understand the necessity, 2 (4%) said they did not have a phone to use, and 7 (14%) said they did not know why they chose not to participate. None of the respondents reported the cost of text messages as a barrier to enrollment.

### Health Department Interviews

Health department staff reported spending between 3 and 60 minutes per day following up on alerts generated through the system. Health systems interacting with respondents receiving the informal texting language indicated that they spent substantially more time (ie, up to 60 minutes) following up on alerts than those interacting with respondents receiving the formal texting language (ie, 3-5 minutes), likely due to a substantial number of false alerts from respondents using colloquial language such as “Aok. All fine here,” rather than using a system-recognized response of “Yes” or “No.”

## Discussion

### Principal Findings

During an outbreak of variant influenza virus associated with swine at agricultural fairs, we successfully deployed a text-based system for illness monitoring among potentially exposed people over a 4-week period. Two H3N2v virus infections were detected among 392 individuals monitored for illness during this outbreak [18], suggesting that text messaging for active surveillance was a valuable tool to complement traditional surveillance methods and enhance detection during this outbreak. Both cases identified through texting were among persons attending fairs with confirmed cases of H3N2v virus infections, suggesting that large numbers of variant influenza cases were not occurring at the other fairs included in this pilot study.

Investigation of novel influenza A virus infection is important for early detection, treatment, and prevention of the spread of influenza viruses with pandemic potential, but passive surveillance methods may miss many infections and underestimate the burden of infection associated with exposure. Multiplier models based on an H3N2v virus outbreak in 2011 estimated that for every pediatric H3N2v virus infection detected and reported to CDC, there were approximately 200 infections in the community; for every one adult H3N2v virus infection, there were approximately 255 infections. Reasons for the gap between reported and actual infections were related to the low likelihood that someone who was ill would seek health care and be tested for a variant influenza virus [22]. While syndromic monitoring lacks the ability to provide direct viral confirmation, active surveillance ensures continued reporting and increases the opportunity for an ill person to interact with health officials and to undergo appropriate testing for detecting variant influenza viruses. In our study, the TIM system was able to detect two additional H3N2v virus infections during an ongoing outbreak and provided a direct way for exposed persons to report symptoms to a health official.

Innovative methods, such as participatory, syndromic surveillance systems for influenza, are increasingly being explored as a means to better capture the burden of influenza at the community level [16,23-26] or in a specific population of interest [17,27]. National and international systems using weekly emails for automated two-way communication between system coordinators and volunteer participants have been implemented in the United States [25], Australia [23], and Europe [26] to capture and track trends in influenza-like illness at a population level. In Australia, another system used SMS text messaging to collect adverse-event data among vaccine recipients [27]. In that initiative, the response rate and timeliness were significantly improved with SMS text messaging in comparison to telephone interviews. Australian health officials also used SMS text messaging to actively monitor responders to an outbreak of avian influenza virus on a poultry farm in 2013. Public health officials found the use of SMS text messaging to be less time-consuming and 2.5 times more cost-effective than conducting telephone follow-up interviews [17].

Systems like TIM could be an important tool in future outbreak investigations. They provide a quick, scalable, and cost-effective method to actively monitor a select group of at-risk people, thereby filling a gap in current surveillance capabilities. Once adopted by a health department, the TIM system can monitor at-risk individuals in an outbreak investigation and be integrated into the outbreak response within hours. As they are intended for detection of illness among a select group of individuals, they complement current influenza surveillance systems that are better adapted for capturing population-level trends and impact [28]. Although the cost of texting was not identified as a barrier in this study, application of this method in less-wealthy populations may benefit from participants being provided phone credit [24].

While the TIM system was well-accepted by both respondents and health departments, we identified three areas where improvements were needed. The first was with enrollment, which varied by fair and ranged from 3% to 86%. The vast majority of households that did not enroll had not heard about the system and those that did enroll did so primarily because it was recommended by the agricultural club coordinator. This highlights the importance of effective communication by people who can reach, and are trusted by, the intended target community. Future deployments of systems like TIM should focus on identifying trusted groups and individuals early and working with them to encourage enrollment. Holding meetings to describe the system and encouraging attendants to enroll during the meeting resulted in higher enrollment, perhaps due to the convenience and personal nature of the recommendation. While strategies to increase enrollment will differ by setting, using personal communication to provide simultaneous rationale and enrollment information appeared to be an effective strategy that could be adapted and applied in most settings.

The second identified area for improvement was testing of symptomatic exposed persons; in our study, only about 20% of ill individuals were tested. Appropriate testing is the only way to confirm infection with a novel influenza virus, as the symptoms are similar to those caused by other respiratory viruses, including seasonal influenza. There are many potential barriers to care-seeking, including lack of motivation to seek influenza testing, the availability of testing at local health care centers or health departments, and the cost associated with visiting a health care provider, especially among people without insurance or with high out-of-pocket health costs. There are several ways to address these barriers and the mechanism depends on the resources and capabilities of the state and local health care systems. In future situations, health officials could consider offering testing for novel influenza virus infections free of charge at health departments or sending clinical staff to collect specimens from ill persons in their homes as a way to address some of these barriers. The latter is a strategy that has been shown to be effective in New York City for longitudinal surveillance of influenza-like illness [16,29]. In that study, home visits were conducted to obtain specimens from persons with reports of illness. Additionally, increased education among exposed persons about the risk of novel influenza infections and the importance of detection also may increase the likelihood of testing.

The third issue identified was with respondent retention. Monitoring for a novel influenza virus requires up to 10 days of monitoring after the last day of exposure, which in the case of a 10-day county fair may necessitate as many as 20 days of monitoring. Maintaining respondents throughout the monitoring period was a challenge in this pilot and only 30% remained enrolled through the end of the recommended monitoring period. We deployed two types of texting language to see if the formality of the language could increase retention. We found that respondents receiving the informal language texts stopped responding sooner than those receiving the formal texting language and fewer remained enrolled through the end of the monitoring period. Respondents of the informal texting language also responded to texts in a conversational tone, often with abbreviations or slang terms, which generated alerts through the system that required unnecessary follow-up by health department staff. This resulted in some health department staff reporting that they spent as much as 60 minutes per day following up on alerts versus 3-5 minutes for those interacting with respondents receiving the formal texting language. We recommend that future deployments of TIM-like platforms should use the formal texting language and consider the optimal length of monitoring that weighs the incubation of variant and avian influenza against the likelihood of attrition among the respondents.

### Limitations

This investigation has certain limitations. First, we conducted it in the middle of an outbreak response and relied on health departments and club coordinators to recruit participants in the middle of a busy fair season. In some situations, recruitment occurred solely through email or delivering flyers, which may have limited the number of people who were aware of the system and its importance. Second, this investigation involved adult participants who responded for the entire household. Other studies also have used this method with success [16]; however, we cannot be certain that the respondent was accurately reporting for all household members. Third, we had a low participation rate to the Web-based survey (14%) and respondents may not have shared the same opinions as those who did not respond. Fourth, our selection of fairs was a convenience sample based on timing and fair attributes. Findings from these fairs may not be representative of all agricultural fairs.

### Conclusions

In summary, we successfully piloted a new text-based monitoring tool for detecting variant influenza virus infections and identified two H3N2v virus infections during an outbreak in Michigan. This pilot demonstrates how text messaging can complement traditional surveillance methods during an outbreak. Future activations of the system should work to improve systematic testing of exposed persons who develop symptoms and should continue exploring methods to improve participation and retention of respondents.

## Appendix

Multimedia Appendix 1 Epidemic curve of influenza A (H3N2) variant virus infections by date of symptom onset and detection method—Michigan, 2016.

## Abbreviations

CDC: Centers for Disease Control and Prevention
H3N2v: influenza A (H3N2) variant
MDHHS: Michigan Department of Health and Human Services
NACCHO: National Association of County and City Health Officials
rRT-PCR: real-time reverse-transcription polymerase chain reaction
SMS: short message service
TIM: text-message illness monitoring
