# Supplementary material for: Text-Based Illness Monitoring for Detection of Novel Influenza A Virus Infections During an Influenza A (H3N2)v Virus Outbreak in Michigan, 2016: Surveillance and Survey
Source: JMIR Public Health Surveill. 2019 Apr 26;5(2):e10842. doi: 10.2196/10842 (PMC6658270; doi:10.2196/10842)
Supplement: Supplementary file 1 [file publichealth_v5i2e10842_app1.docx]

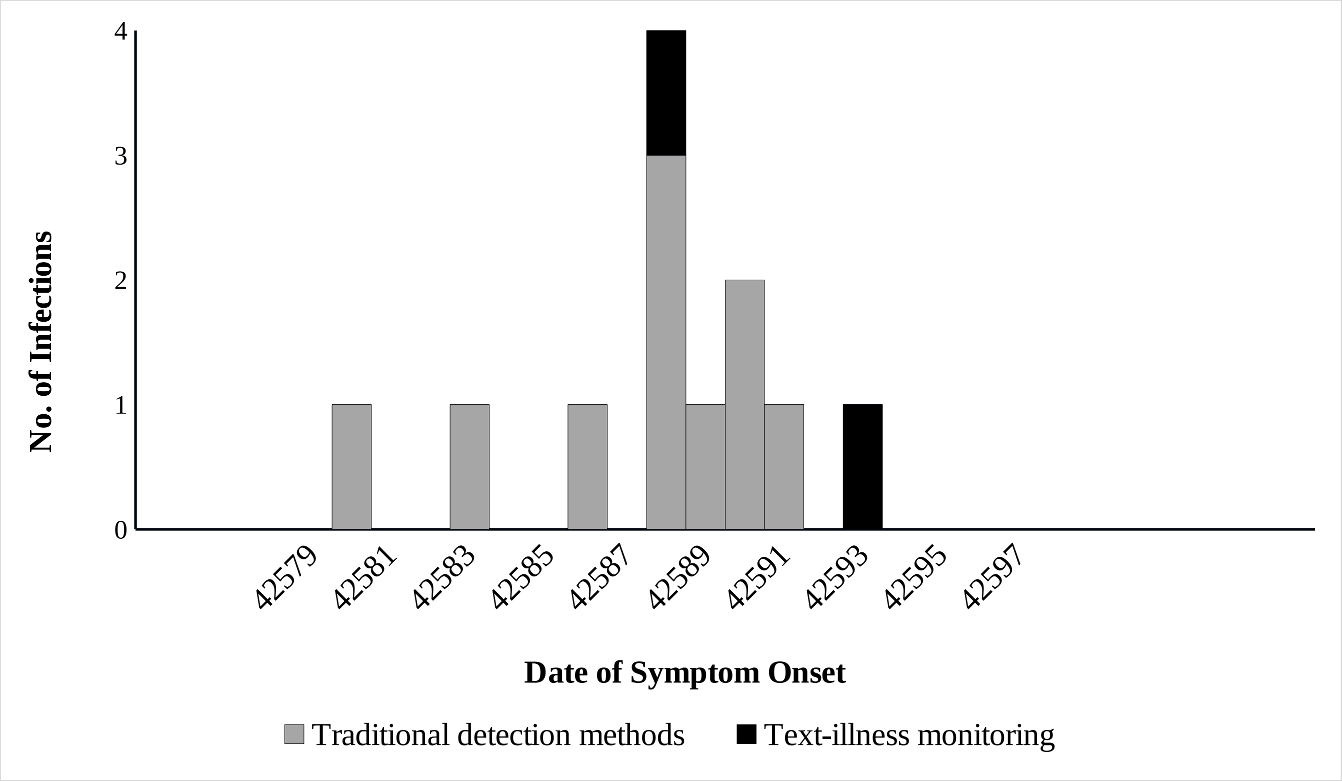
No. of Infections
4
3
2
1
0
42579
42581
42583
42585
42587
42589
42591
42593
42595
42597
Date of Symptom Onset
Traditional detection methods
Text-illness monitoring
